# How Tumors Affect Hemodynamics: A Diffusion Study on the Zebrafish Transplantable Model of Medullary Thyroid Carcinoma by Selective Plane Illumination Microscopy

**DOI:** 10.3390/ijms252413392

**Published:** 2024-12-13

**Authors:** Silvia Carra, Germano Gaudenzi, Giorgia Franceschetti, Maddalena Collini, Laura Sironi, Margaux Bouzin, Luca Persani, Giuseppe Chirico, Giovanni Vitale, Laura D’Alfonso

**Affiliations:** 1 Laboratory of Endocrine and Metabolic Research, IRCCS Istituto Auxologico Italiano, 20100 Milan, Italy; luca.persani@unimi.it; 2 Laboratory of Geriatric and Oncologic Neuroendocrinology Research, IRCCS Istituto Auxologico Italiano, 20100 Milan, Italy; g.gaudenzi@auxologico.it (G.G.); giovanni.vitale@unimi.it (G.V.); 3 Department of Physics “G. Occhialini”, Università degli Studi di Milano-Bicocca, Piazza Della Scienza 3, 20126 Milan, Italymaddalena.collini@unimib.it (M.C.); laura.sironi@unimib.it (L.S.); margaux.bouzin@unimib.it (M.B.); giuseppe.chirico@unimib.it (G.C.); laura.dalfonso@unimib.it (L.D.); 4 Department of Medical Biotechnology and Translational Medicine, University of Milan, 20100 Milan, Italy

**Keywords:** medullary thyroid carcinoma (MTC), selective plane illumination microscopy (SPIM), zebrafish, tumor xenograft, vascular permeability

## Abstract

Medullary thyroid carcinoma (MTC), a rare neuroendocrine tumor comprising 3–5% of thyroid cancers, arises from calcitonin-producing parafollicular C cells. Despite aggressive behavior, surgery remains the primary curative treatment, with limited efficacy reported for radiotherapy and chemotherapy. Recent efforts have explored the pathogenetic mechanisms of MTC, identifying it as a highly vascularized neoplasm overexpressing pro-angiogenic factors. Building on the established benefits of zebrafish embryos, we previously created an in vivo MTC xenograft platform that allows real-time observation of tumor-induced angiogenesis and evaluation of the anti-angiogenic effects of tyrosine kinase inhibitors. In this study, we present a method using selective plane illumination microscopy (SPIM) to characterize vascular permeability in these xenografted embryos. Taking advantage of dextran injections into the blood flow of zebrafish embryos, we found that the diffusion coefficient in embryos grafted with MTC cells was about tenfold lower compared with the same parameter in controls. The results demonstrate the potential of our approach to estimate diffusion parameters, providing valuable insights into vascular permeability changes in MTC-implanted zebrafish embryos compared with controls. Our study sheds light on the intricate vascular biology of MTC, offering a promising tool for future investigations into tumor-induced angiogenesis and therapeutic strategies in diverse neoplasms.

## 1. Introduction

Medullary thyroid carcinoma (MTC) is a rare neuroendocrine tumor (3–5% of all thyroid cancers) originating from calcitonin-producing parafollicular C cells of the thyroid gland [1]. Although the majority of MTCs are sporadic, in 25% of cases MTC occurs in a hereditary form as part of the Multiple Endocrine Neoplasia type 2 (MEN2) syndrome [2].

The clinical course of MTC patients and their survival depend on the stage of the disease at the diagnosis. About half of patients present advanced stage (III or IV) at the initial diagnosis, showing lymph node and distant metastases [3]. The 10-year survival for patients with stages I, II, III and IV MTCs are 100%, 93%, 71% and 21%, respectively [4].

Surgery remains the primary curative treatment for MTC, with limited effects on long-term survival reported for radiotherapy and chemotherapy [3,5]. Patients with metastatic disease may benefit from pharmacological treatment with somatostatin analogs, which can control neuroendocrine symptoms [6,7].

Alterations in the REarranged during Transfection (*RET*) proto-oncogene, encoding a transmembrane receptor of the tyrosine kinase family, represent the most crucial events leading to MTC onset [8,9,10,11,12]. Several tyrosine kinase inhibitors (TKIs) which target RET and different growth factor receptors have been evaluated and approved for clinical use [13,14]. Vandetanib and cabozantinib are the specific drugs used to treat advanced MTC. Vandetanib targets RET, vascular endothelial growth factor receptor-2 and 3 (VEGFR-2 and -3), fibroblast growth factor receptor (FGFR) and epidermal growth factor receptor (EGFR). Cabozantinib targets RET, VEGFR-2 and hepatocyte growth factor receptor (MET) [15,16]. Recently, selpercatinib and pralsetinib have been used to treat different tumors. Their high affinity in targeting RET and low toxicity make these drugs two promising options in treatments for advanced RET-altered MTC [17,18,19,20].

In the last years, substantial efforts have been made to unravel the pathogenetic mechanisms contributing to the onset and progression of MTC. Numerous studies have characterized MTC as highly vascularized neoplasms, demonstrating overexpression of several pro-angiogenic growth factors and their relative receptors [21,22,23,24]. In this context, the hindrance of tumor-induced angiogenesis has been identified as an effective therapeutic approach for patients with MTC.

In recent years, different studies have evaluated vascular morphology and hemodynamics during tumor formation as well as the molecular mechanisms driving tumor vasculature development [25,26,27]. Changes in hemodynamic parameters are strongly correlated to the physical properties of the tumor microenvironment, such as osmotic pressure, and they are fundamental to understand the progression of tumors and their response to therapies.

Animal models able to recapitulate faithfully in vivo the critical features of the interaction between vessels and tumor mass represent a powerful tool to investigate many aspects of vascular biology, making the obtained results translatable into potentially relevant information for human health.

The zebrafish (*Danio rerio*) has emerged as a powerful model organism for studying vertebrate vascular biology, excelling in both developmental and genetic analysis. Its unique characteristics—external fertilization, rapid development, optical transparency and high offspring yield—offer significant advantages over other vertebrate models such as mice. The development of the zebrafish vascular tree has been extensively described and the molecular mechanisms that underlie vessel formation are highly conserved between zebrafish and higher vertebrates [28,29]. Similar to higher vertebrates, the development of the zebrafish vascular tree occurs through both vasculogenesis, which involves the differentiation of endothelial cells from mesodermal precursors followed by the formation of a primitive tubular network, and angiogenesis, the formation of new vessels from pre-existing ones. Given the mechanistic similarities of angiogenesis in embryonic and tumor progression, as well as the high evolutionary conservation of the molecular mechanisms regulating angiogenesis among vertebrates, zebrafish embryos have been extensively used to study tumor vascular biology [30,31]. In addition, many transgenic vascular lines are available, providing the opportunity for direct observation of vascular development in vivo and a detailed analysis of the integrity and functionality of vessels and blood flow in normal or pathological conditions. Moreover, zebrafish has emerged as a suitable model to perform permeability assays. The injection into the blood flow of fluorescent tracers allows the in vivo real-time visualization of blood circulation and vascular tree morphology, the evaluation of the functional integrity of endothelial junctions and the functionality of specific anatomical districts, such as the blood–brain barrier [32,33,34].

The dynamic behavior of the vessel network can be investigated by exploiting the selective plane illumination microscopy (SPIM) technique [35]. SPIM is an advanced imaging technique that uses a thin sheet of light to illuminate a specific plane of a sample through a combination of a cylindrical lens and a microscope objective on the excitation path. The image is collected in wide field at a right angle to the excitation path. In this way, the photodamage and bleaching in out-of-focus planes are reduced and it is possible to achieve a three-dimensional reconstruction of live specimens over extended periods of time at a high resolution. These features make SPIM particularly useful for observing dynamic biological processes, such as embryo development or cell migration, in thick and optically dense specimens. The coupling of the SPIM technique with a charge-coupled device (CCD) camera allows the simultaneous recording of the signal from the whole excited region of the sample. The acquired images are optically confined to individual sections of the sample with a well-defined thickness. The basic idea of SPIM is that the fluorescence is excited only over the plane that is selected, leaving the rest of the sample unexposed and therefore unaffected (no photo-bleaching or photodamage) and collecting the image in wide field orthogonally to the excitation [36].

In this work, we exploited an in vivo platform, based on a xenograft of MTC cells in *Tg(fli1a:EGFP)^y1^* embryos, expressing enhanced green fluorescent protein (EGFP) under the control of the endothelial-specific gene promoter *fli1a*, allowing us to follow the development of the entire vascular tree and the tumor-induced angiogenesis in vivo [37,38].

One of the main advantages of xenotransplantation in zebrafish embryos is the absence of a fully developed immune system at this stage of this procedure, thus no graft rejection occurs [39]. This platform has already allowed us to follow in vivo the tumor-induced angiogenesis, and, taking advantage of the permeability of zebrafish embryos to small molecules dissolved in their culture media, to evaluate the anti-angiogenic activity of specific TKIs [37,40,41]. Here, we coupled permeability assays with SPIM to collect several parameters in vivo that characterize extravasation of a dye in MTC-grafted embryos compared to uninjected controls, providing important information about differences between normal and pathological vascular networks in neuroendocrine tumors.

## 2. Results

### 2.1. System Control Measurements

The functionality of the SPIM setup and the validation of our approach to estimate the diffusion parameters of the fluorescent dye were tested by conducting preliminary control measurements on highly concentrated rhodamine, which diffused slowly in transparent media with varying permeability. To this aim we prepared low melting agarose (Sigma-Aldrich, Merck KGaA, Darmstadt, Germany) solutions at different mass percentage concentrations (1%, 2% and 5%) and we let them cool down and solidify in rectangular quartz cuvettes (to reduce optical mismatch).

When they were solidified, we positioned the cuvette inside the immersion chamber, and we injected a small drop of highly concentrated rhodamine (>2 mM) inside the solidified agarose in order to investigate the diffusion of the dye. Immediately after injection, image stacks of more than 700 frames were acquired every 5 s.

**Figure 1:**
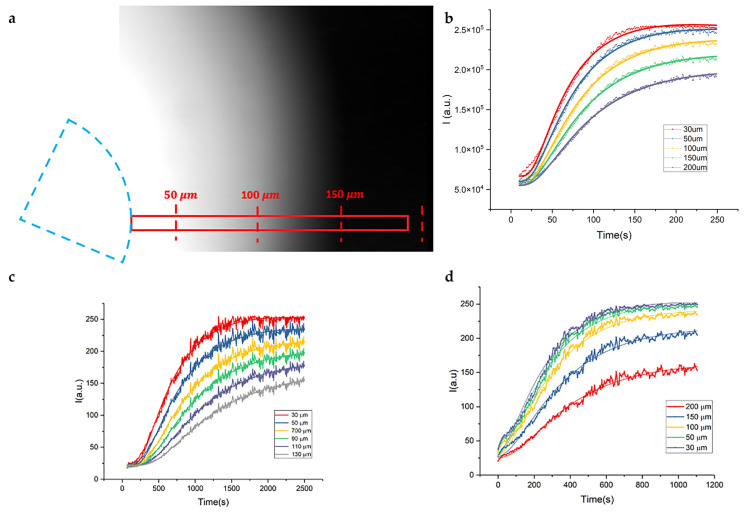
Analysis of the time stack of images acquired for 5 min (5 s time steps) after the injection of very concentrated rhodamine in solidified agarose. (**a**) One frame acquired a few seconds after injection. The dashed blue hemi-circle indicates the cross-section of the needle for injection. The red rectangle represents one of the ROIs over which the intensity profile was measured versus time at different distances from the injection point. (**b**–**d**) Intensity profiles measured on ROIs at increasing distance from the injection point (in the legend), as described in the legend, from samples at different agarose concentrations (1, 2 and 5%, respectively), together with their best fit to Equation (4) (continuous line).

Figure 1a shows, as an example, an image acquired 5 min after injection, and the red rectangle in the image represents one of the regions of interest (ROIs) over which the intensity profile was measured versus time. Different ROIs were chosen at increasing distances from the injection point (from about 30 μm to about 200 μm), to monitor the diffusion of the dye. The intensity profiles measured in the ROIs are shown in panels b, c and d of Figure 1. These profiles, derived from samples with different agarose concentrations (1%, 2% and 5%, respectively), are presented along with their best fit to Equation (4) (see Section 4) in Figure 1. From the global fitting of the data acquired at increasing distances from the injection point, it was possible to estimate the value of the diffusion coefficient of the dye in the different samples, as reported in Table 1.

Most of the works in the literature have focused on the diffusion of small dyes (rhodamine and methylene blue [43] or phthalocyanine dye [44], with molecular weight ≃600D) for which the diffusion coefficient is about $D≃150\frac{\mu m^{2}}{s},$ about 10 times larger than the one expected here for the larger MW dextran. If we assume a simple mass scaling law for the diffusion coefficient, $D\propto M^{-\frac{1}{3}}$, given the $≃10^{4}$ times larger molecular weight of dextran compared with the simple dyes used [43,44] we would expect a value $D≃7\frac{{\mu m}^{2}}{s}.$ However, diffusion in gels is anomalous [45] and a shallower scaling law is expected above a critical size. Altogether, the values reported in Table 1 can be considered in good agreement with the literature [43].

**Figure 2:**
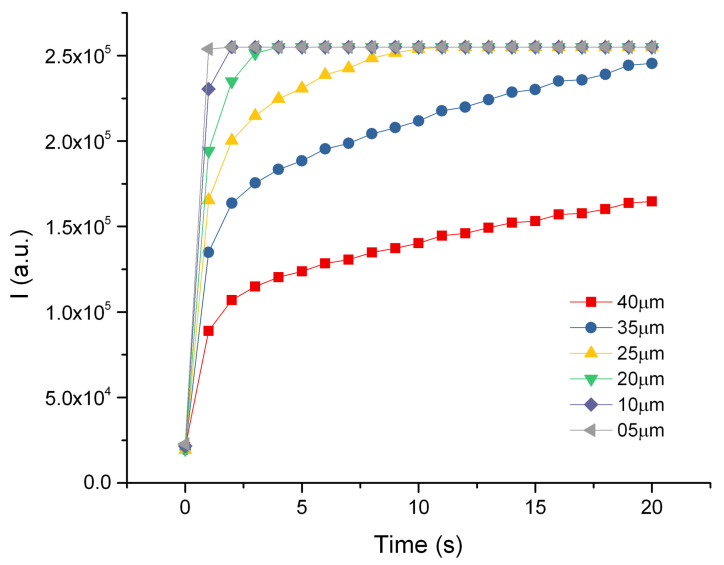
Intensity profiles of diffusing rhodamine in jellified 1% agarose solution. Different intensity profiles are recorded at different distances from the injection point as a function of time.

A crucial parameter of this experiment was the distance of the field of view from the injection point. The closer the injection point, the shorter the time needed for the signal to reach the saturation point. Our setup and detection unit can follow the details of the kinetics with high spatial and time resolution, as shown in Figure 2 for the case of a 1% agarose gel injected only few micrometers from the field of view. As Figure 2 shows, the intensity profiles evaluated at increasing distances from the injection point reach their saturation value in just a few seconds. The rapid linear increase at very short times is due to pressure effect right after the injection.

### 2.2. Zebrafish Morphology and Diffusion Analysis by SPIM Dynamic Microscopy

**Figure 3:**
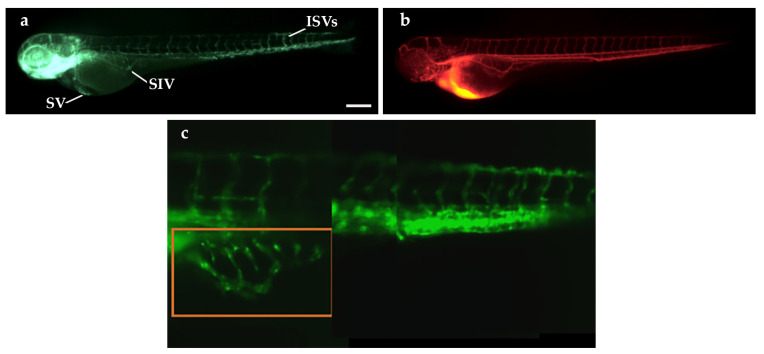
Microangiography assay in *Tg(fli1a:EGFP)^y1^* embryos at 3 dpf and 3D reconstruction of the vasculature. (**a**) Fluorescence image of a 3 dpf *Tg(fli1a:EGFP)^y1^* embryo, showing the entire vascular tree in green. (**b**) The red emission of the dextran rhodamine injected into the blood flow of the same embryo by microangiography. This image was taken immediately after the microangiography. SV: sinus venosus; SIV: subintestinal vein; ISVs: intersegmental vessels. Scale bar: 100 µm. (**c**) Reconstruction of the vasculature at the level of yolk and tail of a 3 dpf embryo made by means of SPIM microscopy. The red-boxed area corresponds to the SIV region. The image is the stitching of three sheet images. Embryos are shown anterior to the left.

Basic morphological studies on 3-days-post-fertilization (dpf) zebrafish *Tg(fli1a:EGFP)^y1^* embryos were performed to verify the ability of our SPIM setup to visualize the vascular system of the embryos. We first exploited the SPIM microscope to obtain a 3D reconstruction of the vascular tree, with a particular interest in the subintestinal vein (SIV) plexus region (Figure 3c).

To analyze in vivo the diffusion of dextran rhodamine, we performed a microangiography on 3 dpf *Tg(fli1a:EGFP)^y1^* embryos in the sinus venosus (SV, Figure 3a,b). Immediately after the injection, we acquired some representative images with a fluorescence stereomicroscope that confirmed the spread of the injected dye into the entire vascular network of the embryo (Figure 3b). Immediately afterwards ($t_{0}≃300\;s),$ the embryo was inserted vertically into a fluorinated ethylene propylene (FEP) tube.

Therefore, the time delay between the injection and the starting of the fluorescence kinetic was of the order of $t_{0}≃300\;s$. Following the procedure established for the measurements of the diffusion coefficient of rhodamine in agarose, we positioned the sample in the focal plane of the SPIM setup, and, by exciting the endothelial EGFP fluorescence by means of the 488 nm Argon ion laser line, we focused about 10 µm–20 µm from the center of the SIV plexus, far away from the injection site. We then acquired stacks of images of the diffusing dextran over time. Dextran fluorescence was excited by the 514 nm Argon ion laser line.

**Figure 4:**
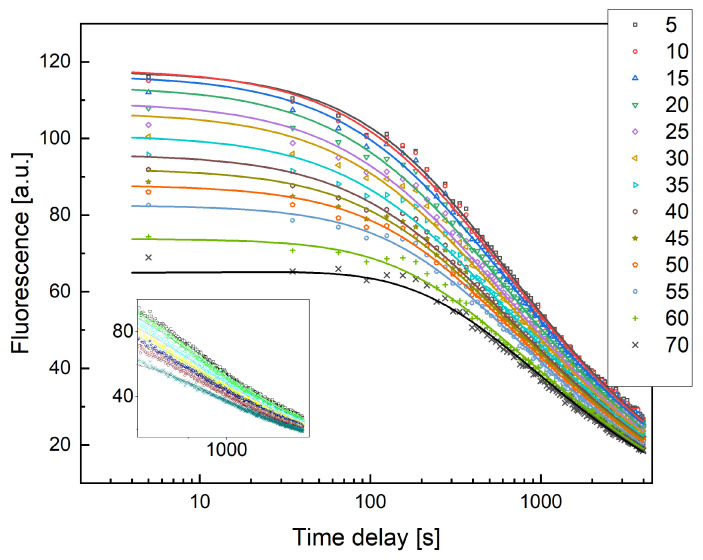
Fluorescent dextran intensity profiles versus time measured at increasing distances from the SIV plexus for a control zebrafish embryo, as shown in the legend. Solid lines are the best fit of the data to Equation (4), performed to determine the value of the diffusion coefficient D, kept as a shared global parameter. The time delay between the injection and the observation was set to $t_{0}≃320\;s.$ The solid lines are the best fit to the data with best fit value of the diffusion coefficient $D=12\pm 0.2\frac{{\mu m}^{2}}{s}$.

Figure 4 reports an example of the resulting intensity profiles over time, sampled on ROIs selected at increasing distances from the SIV plexus (at 5 µm steps) on the control zebrafish embryos. As the distance of the ROI from the SIV increases, the value of the initial fluorescence intensity decreases. This is consistent with the diffusion of the dye from a confined source as can be gained from the rapid exponential decay reported in Equation (4). The profiles have been smoothed through a Savitzky–Golay filter and globally fitted to Equation (4) to determine the value of the diffusion coefficient. A single diffusion component faithfully recapitulates the data (Figure 4), with a diffusion coefficient $D=12\pm 0.2\frac{\mu m^{2}}{s}$ averaged over multiple experiments (>3) on six different living samples per condition. The experimental delay time for the experiments on zebrafish embryos without tumor cell implant (controls) was $t_{0}≃320\;s$.

### 2.3. Diffusion Analysis on MTC Xenotransplanted Zebrafish Embryos

To analyze vascular permeability defects in a pathological condition, taking advantage of tumor xenograft assays in zebrafish, we compared diffusion parameters between embryos implanted with MTC cells and controls.

**Figure 5:**
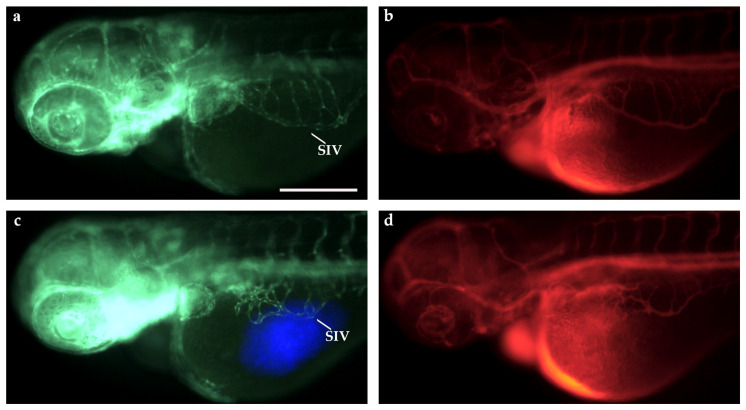
Microangiography assays in TT-xenografted and control *Tg(fli1a:EGFP)^y1^* embryos at 3 dpf. Fluorescence images of 3 dpf *Tg(fli1a:EGFP)^y1^* embryos, injected at 2 dpf with PBS as control (**a**,**b**) and with blue fluorescence stained TT cells (**c**,**d**) at the level of the subperidermal cavity near the SIV, and by microangiography (**b**,**d**). The dextran tetramethylrhodamine signal is red. Grafted larvae showed vessels in green that sprout from the SIV towards the xenograft in blue (**c**). Embryos are shown anterior to the left. SIV: subintestinal vein. Scale bar: 100 µm.

At 48 h post fertilization (hpf), *Tg(fli1a:EGFP)^y1^* embryos were xenotransplanted with TT cells, a stabilized cell line derived from human MTC. Control embryos were injected with only PBS in place of tumor cells, as described in the Section 4. Xenograft was performed in the subperidermal cavity, in proximity of the SIV plexus. Microangiography experiments were performed at 24 h post implantation on xenografted embryos, as described for the control embryos. At 24 h post injection, xenografted embryos showed new vessels induced by tumor cells that sprout from the SIV (Figure 5c,d), in marked difference from the control embryos (Figure 5a,b).

**Figure 6:**
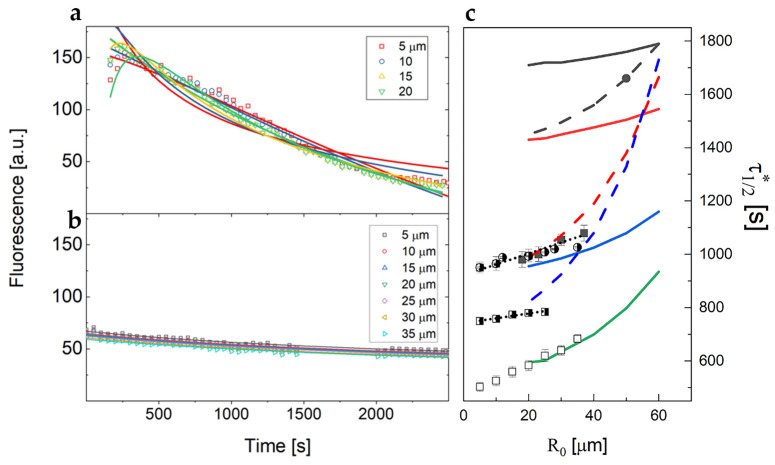
Examples of fluorescent dextran intensity profiles over time measured on different xenografted zebrafish samples at different distances from the injection point. Solid lines are the best fit with $D=1.9\pm 0.22\frac{\mu m^{2}}{s}\;$ (**a**) and $D=0.82\pm 0.02\frac{\mu m^{2}}{s}\;$ (**b**). (**c**) Trend of the half value time $\tau_{\frac{1}{2}}$ as a function of the distance, $R_{0}$, of the observation volume from the injection volume for control embryos (open squares) and for three different xenotransplanted embryos (filled squares, half-filled squares and circles). Solid lines correspond to the simulations for D = $10\frac{{\mu m}^{2}}{s}$ and $t_{0}=1800\;s,\;1200s,\;600\;s,\;300\;s$, from top to bottom. The dashed lines correspond to the simulations for D = $4\frac{{\mu m}^{2}}{s}$ and $t_{0}=1200\;s,\;600\;s,\;450\;s$, from top to bottom. The dotted lines are the best fit linear trends to the data.

In the case of xenografted embryos, we observed significantly higher variability (see Figure 6a,b) in the diffusive properties of the fluorescent dextran compared with controls (Figure 4). In fact, widely different intensity kinetics were found for the xenografted embryos (Figure 6a,b) with no marked dependence on the ROIs in the image, each placed 5 µm apart. For the control embryos, the fluorescence kinetics were highly reproducible and show the expected dependence on the distance of the ROI from the vessel.

These distinct behaviors contributed to increasing the variability in the retrieved diffusion coefficient values. When the profiles were smoothed through a Savitzky–Golay filter and then globally fitted to Equation (4), the average value of the diffusion coefficient (over four different zebrafish embryos) was D = 1.0 ± 0.1 μm^2^/s, with an uncertainty of 10%. This average value of the dextran diffusion coefficient found in xenografted zebrafish samples, also taking into account the large variability observed, is more than tenfold lower compared with the value of the diffusion coefficient previously measured in controls.

As a second estimate of the diffusion parameters, we plotted the fluorescence half time $\tau_{\frac{1}{2}}$ (i.e., the time at which the fluorescence intensity is reduced to half of its initial value) versus the distance from the SIV of the ROI on which the intensity profiles were evaluated. The half time $\tau_{\frac{1}{2}}$ is an effective value since we have no direct access to the maximum of the curve describing the concentration as a function of time. This is due to the time delay between the injection and the start of the observation, parametrized by $t_{0}$. Experimentally, we found that $\tau_{\frac{1}{2}}$ depends linearly over the ROI–SIV distance, as shown in Figure 6c, even though it is not a direct proportionality. As discussed in Appendix A, the measured half-decay time has a complex relation with the actual half-decay time; that is, the time at which the concentration drops at half the value of the maximum reached at a distance $R_{0}$ from the injection volume (see Figure A4). Nevertheless, the parameter $\tau_{\frac{1}{2}}$ can be exploited in two ways. First of all, the slope $v≃\frac{R_{0}}{\tau_{\frac{1}{2}}}$ can be heuristically taken as the velocity (Equation (5), see Section 4) of the dextran concentration wave, diffusing through the SIV plexus. The value of the wave speed $v=0.10\pm 0.01\frac{\mu m}{s}$ that we find for control zebrafish embryos can be converted into a second independent estimate of the diffusion coefficient through Equation (6) (see Section 4). As a matter of fact, as shown in Figure A2 and Figure A3, the Equations (5) and (6), are equivalent to a generalized diffusion process where the mean square displacement $<\Delta r^{2}>$ is given by:

(1) $$<\Delta r^{2}>≃R_{0}^{2}≃4\frac{D_{v}}{f\left(D_{v}\right)}\tau_{\frac{1}{2}}$$

For example, if we apply Equation (1) to the data collected on control zebrafish embryos (see Figure 6c) we estimate the value $D_{v}≃7\frac{\mu m^{2}}{s}$ for the diffusion coefficient (at $\tau_{\frac{1}{2}}≃700\;s$), in fair agreement with the best fit diffusion coefficient obtained by a global fit of the model (Equation (4)) to the data (see Figure 4, $D≃12\frac{{\mu m}^{2}}{s}$).

Secondly, as shown in Figure A4, the trend of $\tau_{\frac{1}{2}}$ on $R_{0\;}$ depends sensibly on the value of the delay time, $t_{0}$. In this way, we can independently estimate the delay time $t_{0\;}$ in the observation of the diffusive motion in zebrafish that results $t_{0\;}≃$300 s (see Figure 6c, green line and open squares, for an example).

For the xenografted embryos, a variability similar to that observed for the diffusion coefficient was found for the trend of the half-decay time, $\tau_{\frac{1}{2}}$, of the fluorescence traces on the distance $R_{0\;}$ of the observation volume from the SIV plexus. Additionally, for the xenografted embryos we can independently estimate the delay time $t_{0\;}$ from the trend of $\tau_{\frac{1}{2}}$ as a function of $R_{0\;}$. In this case, this analysis, whose result is summarized in Figure 6c, gives $t_{0\;}≃$600 s for the xenografted embryos, sensibly longer than the result of the control embryos data and the known experimental conditions, which is $t_{0}\;≃$ 300 s.

## 3. Discussion

Angiogenesis has been identified as a key mechanism in the progression of MTC. The hindrance of tumor-induced angiogenesis has been identified as an effective therapeutic approach. Currently, the available therapeutic strategies target RET and different growth factors and their receptors involved in angiogenesis [21,22,23,24]. The pressing need to identify novel therapeutic strategies, together with the absence of preclinical models that thoroughly characterize the structure and permeability of vessels within the tumor vascular plexus for this type of cancer, has driven us to shed light on the hemodynamics of tumor-induced vessels for the first time.

It is well documented that multiple factors can influence internal and peritumoral blood flow in solid tumors, which is often heterogeneous and compromised, promoting hypoxia and the spread of tumor cells, as well as limiting drug delivery [46,47]. Indeed, altered adhesion between endothelial cells in tumor vessels that leads to the formation of unstable and hyper-permeable vessels [48], together with the mechanical pressure that tumor and stromal cells exert on immature, intricate and highly dense tumor vessels, promote the leak of fluids. The resulting increase of interstitial fluid pressure, which is often not solved for the presence of an inadequate lymphatic system, is considered a barrier for the movement of drugs towards tumor cells [49,50,51,52,53,54].

At a technical level, our study aimed to develop a portable SPIM setup to describe, in vivo and in real-time, extravasation changes in MTC-grafted embryos compared with a control with no engraftment. In particular, we developed a method based on the injection of dextran rhodamine (clearance time of about 30 min) into the zebrafish blood flow and on the subsequent observation of the extravasation process (at time steps of 5 s) by SPIM.

In this context, microangiography experiments performed in zebrafish embryos coupled with acquisitions carried out with our SPIM system gave us the opportunity to characterize the extravasation in health and pathological conditions.

In vivo SPIM microscopy allowed us to achieve the sectioning of a three-dimensional sample similar to that obtained by confocal microscopy without the use of a pinhole along the detection path. In the SPIM technique, the sample is illuminated from the side with a sheet of light, and the light emitted or scattered by the illumination layer is recorded by a camera orientated on an axis parallel to the sheet. The primary advantage of a SPIM setup lies in the utilization of a thin sheet of light to excite fluorescence in the sample and capture images in a wide-field modality. In our setup, the light sheet is approximately 8 μm thick and extends over an area of about 300 μm×300 μm. Compared to confocal microscopy, SPIM offers two main advantages: it minimizes the effects of photobleaching and phototoxicity on the sample since only the illuminated plane contributes to the fluorescence signal, leaving fluorophores outside the sheet unexcited; additionally, the illumination and detection paths are positioned orthogonally, effectively decoupling the excitation and emission paths. This allows one to choose different excitation and emission systems, with varying magnifications, objective NA and working distances. Moreover, while confocal microscopy typically acquires data through a raster scan path, SPIM captures the entire illuminated sheet simultaneously using a pixelated detector (CMOS or CCD camera). Therefore, the acquisition process is optimized and the temporal resolution is given by the camera frame rate. The only disadvantage of this technique is related to the non-transparency of some samples, which induce a degradation of the shape of the light sheet due to the high scattering signal. In this work, taking advantage of microangiography in zebrafish embryos, we evaluated and compared the velocity and the amount of dye extravasation in normal and pathological conditions induced by the implantation of MTC cells.

**Figure A1:**
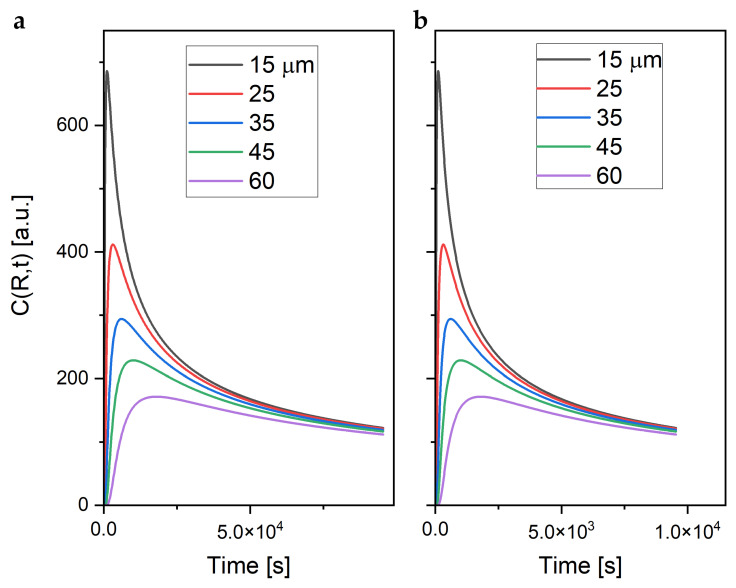
Simulations of the time trend of the concentration for increasing values of the distance, R, between the injection and observation sites. The values of the parameter R are shown in the legends. Panels (**a**) and (**b**) refer to the case of $D=0.1\frac{\mu m^{2}}{s}$ and $D=1\frac{\mu m^{2}}{s}$, respectively. $R_{0}=0,\;t_{0}=0$.

**Figure A2:**
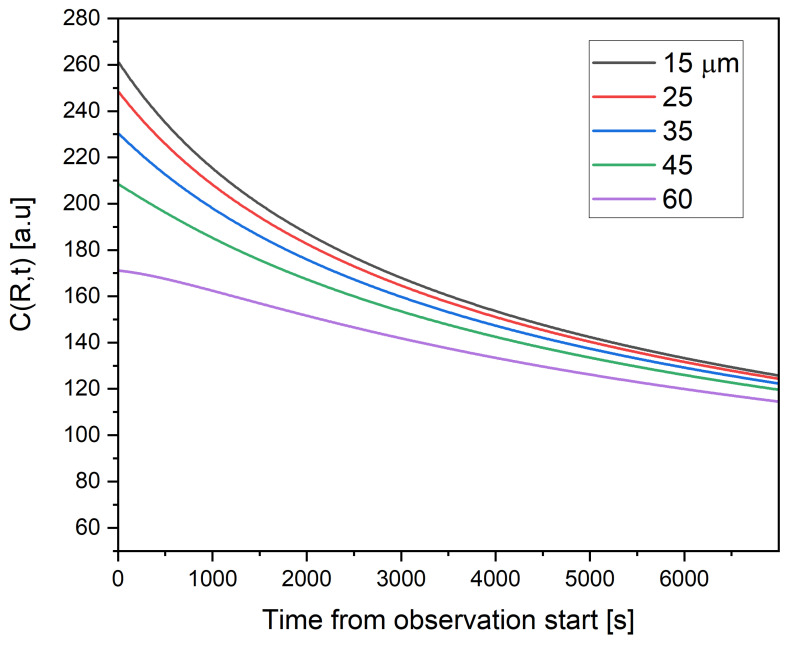
Simulation of the concentration (and fluorescence) decay as a function of time for the case in which the injection was administered at t = 2000 s prior the start of the observation. The diffusion coefficient was D = 1$\frac{{\mu m}^{2}}{s}$.

**Figure A3:**
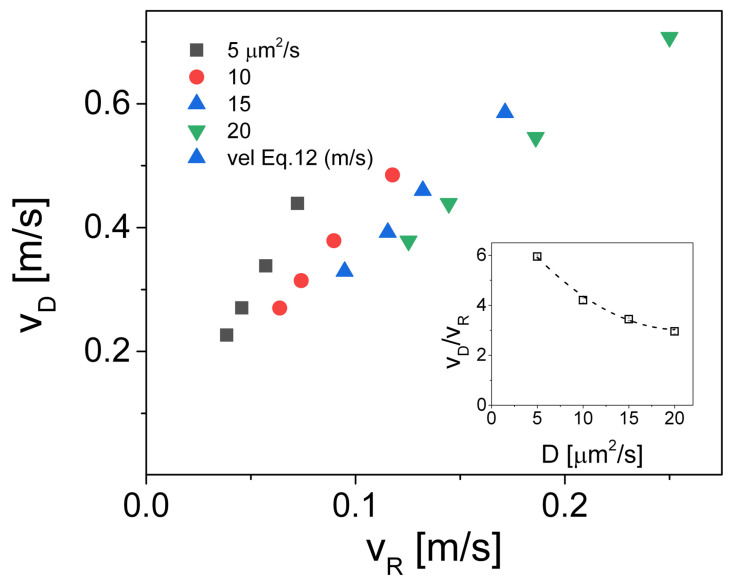
Simulation of the relationship between the two estimates of the spread of the concentration wave, $v_{R}$ and $v_{D}$, as a function of the diffusion coefficient. The inset reports the value of the slope $\frac{dv_{D}}{dv_{R}}$ as a function of D. The dashed line is the best fit polynomial function: f(D) = 0.013 D^2^ − 0.51 D + 8.14.

**Figure A4:**
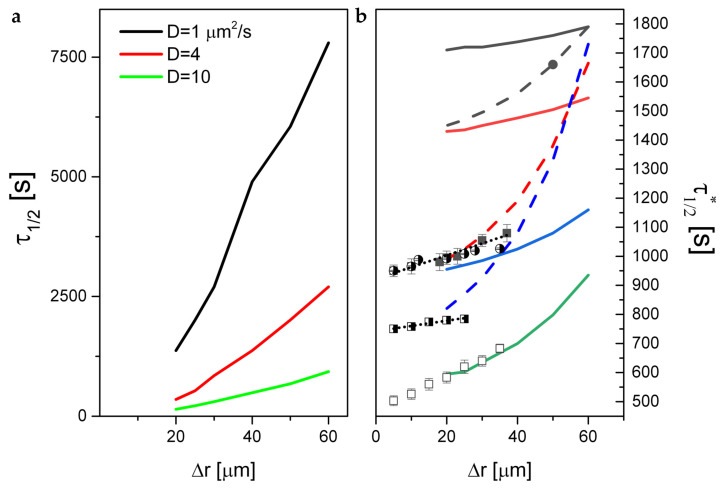
Simulations of the half-decay time as a function of the distance of observation from the injection volume. (**a**) Graph reports the study of $\tau_{\frac{1}{2}}$ as a function of the diffusion coefficient for D = $1\frac{{\mu m}^{2}}{s}$, $4\frac{{\mu m}^{2}}{s}$ and $10\frac{{\mu m}^{2}}{s}$, as reported in the legend. (**b**) Graph reports the effect of the delay time $t_{0}$ and the diffusion coefficient on the experimental value $\tau_{\frac{1}{2}}^{*}$. Solid lines correspond to the simulations for D = $10\frac{{\mu m}^{2}}{s}$ and $t_{0}=1800\;s,\;1200\;s,\;600\;s,\;300\;s$, from top to bottom. The dashed lines correspond to the simulations for D = $4\frac{{\mu m}^{2}}{s}$ and $t_{0}=1200\;s,\;600\;s,\;450\;s$, from top to bottom. The solid and open squares are experimental data taken on xenotransplanted and control zebrafish. The data were collected with an estimated delay time of 300 s and 380 s for the xenotransplanted and control zebrafish, respectively.

We have chosen to focus our analyses on the SIV plexus, far away from the SV microinjection site. In the SIV region, important remodeling events take place during zebrafish development, and so it could be considered a good example of a vascular district where endothelial junctions are prone to plasticity. In zebrafish, the SIV has a diameter ranging from 10 µm to 30 µm, therefore it can be easily imaged with our SPIM setup. In a first set of experiments, we analyzed the physiological leakage of dextran from the SIV plexus. The diffusion of dye extravasating from the vessels leads to an increase of the fluorescence intensity that then decreases over time (see also Figure A1). The larger concentration found near the SIV persists over a wide time period (see Figure 4). Subsequently, we used our SPIM system to analyze tracer diffusion in both the absence and presence of an MTC engraftment in zebrafish embryos.

Due to the procedure of tracer injection, we were not able to follow the very first events after the injection, even though by SPIM imaging we acquired information on a wide field of view, comprising also the region of the injection. Therefore, it was not possible to directly identify changes in the extravasation rate right after the injection. However, we have an indirect indication of the fact that extravasation occurs differently in the xenotransplanted zebrafish embryos than in controls by studying the relation between the position of the observation region of interest and the half time decay of the fluorescence decay (Figure 6c). As a matter of fact, a delay in the extravasation would result in a shift of the whole fluorescence trace towards longer times, resulting in an effective increase in the best fit value of the delay parameter $t_{0}.$ Indeed, we find that there is a systematic increase in the measured delay time for the xenotransplanted embryos (600 s, as per Figure 6c, compared with the experimental 300 s) with respect to the controls without implant.

The changes in the effective diffusion coefficient, as can be gained from the fit of the decay of the fluorescence signal with time (see Figure A1 for example), can instead be related to the microscopic state of the tissue around the vessel. From our analyses we found that the diffusion coefficient in embryos grafted with MTC cells was about tenfold lower compared with the same parameter in controls. Thus, a lower velocity of tracer diffusion, directly proportional to the diffusion coefficient, has been found in xenografted embryos than in controls. A possible explanation of this observation is that it is due to the increase of interstitial pressure in the peritumoral region of grafted embryos, as a consequence of the above-described factors, linked to the hyper-permeability of tumor vessels. Notably, we analyzed the tracer diffusion at 24 h after the implantation of MTC cells, when tumor-induced vessels are already formed (as can be seen in Figure 5). Interestingly, these alterations in the interstitial pressure have been already described in other xenograft models [49,55].

The flow pattern in tumor tissues is complex and the analysis of the flow dynamics within the tumor is technically challenging in vivo. The complex interplay between extravasation, permeability, vascular heterogeneity and drug transport in MTC remains poorly understood. Thus, the deeper understanding of tumor vascular features that we described in this zebrafish study for MTC not only sheds light on the intricate biology of MTC but also paves the way for treatments that may significantly impact patient outcomes. As several studies have identified, the use of anti-angiogenic therapy as an effective strategy [13,14] and also the ‘normalization’ effect of some agents on vessel remodeling may improve the perfusion, oxygenation and the efficacy of specific tumor therapies.

Hopefully, our zebrafish platform coupled with our portable SPIM system might represent an innovative model to analyze the effect of specific drugs able to normalize peritumoral vasculature. In this context, it could be interesting to test the impact of newly introduced drugs for advanced MTC treatment, such as selpercatinib and pralsetinib, on vascular integrity using zebrafish.

Moreover, our model can be exploited in future studies on other neoplasms to characterize the properties of their tumor vessels and to identify new therapeutic strategies aimed to stabilize endothelial structures.

## 4. Materials and Methods

### 4.1. Two-Dimensional Portable SPIM Setup

**Figure 7:**
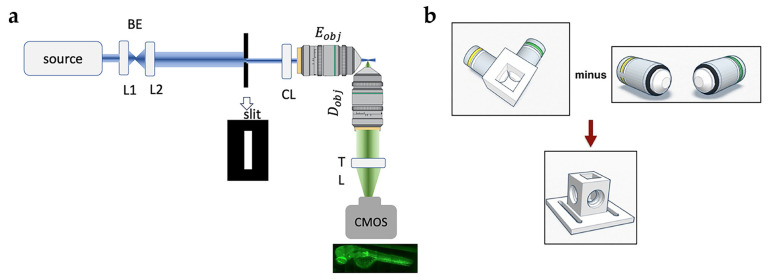
(**a**) Schematics of the main parts constituting the portable SPIM setup employed in this study. The source beam is expanded by the beam expander (BE) composed of lenses L1 and L2, filtered by the slit and shaped as a light sheet by the cylindrical lens (CL) and the excitation objective (Eobj). The light is collected at a right angle with respect to the excitation by a 4f system composed of the collection microscope objective (Dobj) and the tube lens (TL) and the image is created on a CMOS camera. (**b**) Schematics of the 3D-printed immersion chamber.

Our 2D portable SPIM setup is sketched in Figure 7a. The overall size is 50 × 35 cm. This comprises all the optical parts and the detector. The source is coupled in air to the entrance beam expander (BE in Figure 7a). For the experiments in this report, we used a multi-line Argon laser (Spectra Physics, 2050, broadband) that impinges on a 2X beam expander (BE, f_1_ = 25 mm, f_2_ = 50 mm) that magnifies the excitation profile and hits a rectangular slit. A cylindrical lens (CL, f_3_ = 50 mm), placed after the slit, is coupled with the excitation objective (Olympus UMPLFLN 10X, water immersion, NA = 0.3, 3.5 mm working distance), to generate a sheet-like profile used to illuminate the sample. Wide-field detection occurs orthogonally to the excitation light sheet. An objective-tube lens couple (Olympus 2MPLFLN 20X, water immersion, NA = 0.5, 1.5 mm working distance, TL lens focal length f = 70 mm) creates an image on a CMOS camera (iDS; 4.92 megapixels CMOS sensor, U1-1240SE). This small sensor is easily mountable on our portable SPIM setup. It offers a resolution of 2560 × 1920 pixels (2.20 μm pixel size), and a pixel a frame rate of 6.3 fps. The CMOS sensor is intrinsically more sensitive than the classic CCD camera. However, in order to increase the sensitivity at the expense of the spatial resolution, we have re-binned the frame 4 times. The camera is connected to the computer thanks to a USB port, and it is driven through the Micromanager plugin in Fiji [56].

The sample in our setup was placed in a 3D-printed immersion chamber to provide optical index matching and to keep the excitation and detection objectives in an orthogonal geometry, as represented in the sketch in Figure 7b.

For the calibration and the diffusion measurements we exploited different lines of a multi-line Argon laser system (Spectra Physics 2025). The proper wavelength to excite the samples was selected through an interferential small band filter. In order to excite the GFP expressed in the endothelium of the embryos we employed the 488 nm line (350 mW output power), while the 514 nm line was used to excite the rhodamine–dextran solution (420 mW output power).

The Point Spread function (PSF) of the optical system was measured by recording images of 1 µm size fluorescent latex beads. Stacks of images were acquired under minimum camera exposure conditions to ensure the presence of the same particle on multiple frames. Morphological fluorescence images of zebrafish embryos were captured at 488 nm corresponding to the excitation of the GFP in the embryo’s endothelium, and collecting wide (field of view, FOV, of 880 × 700 µm^2^) zebrafish sections in a single image thanks to a tube lens of reduced focal length (70 mm).

### 4.2. Characterization of the Optical System: Thickness of the Light Sheet

**Figure 8:**
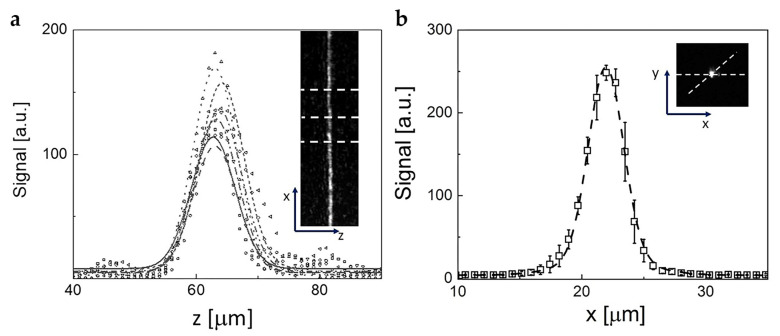
(**a**) Example of the profiles of the cross section of the light sheet image acquired by means of a reflecting mirror (640 × 512 pixels, 880 µm × 700 µm). The inset reports the image of the sheet cross-section on which the dashed lines indicate only a few of the sections that are analyzed in the main panel. The lines in the main panel are the best fit of the profiles to a Gaussian function with an average fitting FWHM = 7.8 ± 1.3 µm. (**b**) Example of the average cross-section of a single 1 μm fluorescent bead imaged in the light sheet used to determine the value of the PSF of the setup (640 × 512 pixels, 880 × 700 µm^2^). The dashed line is the best fit Gaussian function to the data with FWHM = 3.2±0.4 μm. The inset shows an example of the image of a microsphere in the light sheet.

To obtain the best possible optical sectioning, it is necessary to align the SPIM microscope daily and to characterize the shape of the beam and the thickness of the light sheet before starting the acquisitions. The alignment of the system must be checked in excitation and in detection. In the latter case, we checked that the sample plane of the detection objective ($D_{obj\;}$ in Figure 7) coincided with the excitation light sheet. For the excitation path, on the other hand, one needs to check the optical sectioning capability, which is determined by the thickness of the light sheet. To measure it, we used a reflecting mirror inserted at the focal plane and displaced by 45 degrees from the direction of the light sheet. By moving the mirror orthogonally to the direction of propagation, it is possible to scan the light profile along its whole length. If the system is well aligned, the shape of the profile will not depend upon the position of the mirror over a wide field of view. An example of an image of the light sheet obtained by means of the reflecting mirror is shown in Figure 8a.

To evaluate the thickness of the light sheet, each line of the image the beam profile was measured and then fitted to a Gaussian function. The FWHM (Full Width Half Maximum) of the profile was taken as a measure of the width of the light sheet. The thickness of the light sheet was measured daily, yielding an average FWHM = 7.8 ± 1.3 µm. The error represents the daily variance of the thickness. The corresponding Rayleigh range is 400 ± 120 μm.

### 4.3. Characterization of the Optical System: Point Spread Function

The resolution of the system was estimated by measuring the PSF of the setup. Fluorescent latex beads of 1 µm diameter were spread on a glass slide and dried. Within each image, regions of interest were selected where single particles were present, as judged from the integrated intensity of the spot. From the Gaussian fit of the intensity profiles over a hundred particles, obtained by radially dissecting the three-dimensional peaks, it was possible to estimate the radial dimension of the PSF, finding that PSF_x,y_ = 3.2 ± 0.4 μm. An example of the collected images and of the resulting intensity profile is reported in Figure 8b, together with the corresponding Gaussian fit.

### 4.4. Cell Culture

The TT cell line was kindly provided by Prof. Lips (Utrecht, the Netherlands). Cells were maintained at 37 °C in 5% CO_2_ and cultured in T75 flasks filled with 10 mL of F-12K Kaighn’s modification medium (Gibco™ Thermo Fisher Scientific, Waltham, MA, USA). The medium was supplemented with 10% heat-activated fetal bovine serum (FBS) (Invitrogen™ Thermo Fisher Scientific, Waltham, MA, USA), 10^5^ U·L^−1^ penicillin/streptomycin (EuroClone™, Milan, Italy). Cells were harvested by trypsinization (Trypsin 0.05% and EDTA 0.02%; Sigma–Aldrich, Merck KGaA, Darmstadt, Germany) and resuspended in complete medium, then counted through an optical microscope using a standard hemocytometer before plating. The cells used in all experiments were below 5 passages.

### 4.5. Zebrafish Line and Maintenance

Embryo and adult zebrafish (Danio rerio) were raised and maintained according to Italian (D.Lgs 26/2014) and European laws (2010/63/EU and 86/609/EEC). Embryos were staged according to morphological criteria [57]. All experiments were performed using zebrafish embryos belonging to the transgenic line Tg(fli1a:EGFP)^y1^, expressing EGFP under the control of the endothelial-specific gene promoter fli1a, allowing in vivo visualization of the entire vascular tree [58].

Zebrafish embryos obtained from natural spawning were raised and maintained according to established techniques [59]. Starting from 24 hpf, embryos were cultured in fish water (0.1 g/L NaHCO_3_, 0.1 g/L Instant Ocean, 0.192 g /L CaSO_4_•2H_2_O) containing 0.003% PTU (1-phenyl-2-thiourea; Sigma–Aldrich, Merck KGaA, Darmstadt, Germany) to prevent pigmentation and 0.01% methylene blue to prevent fungal growth.

### 4.6. Tumor Xenograft and Microangiography Experiments in Zebrafish

At 2 dpf, embryos were anesthetized with 0.016% tricaine (Ethyl 3-aminobenzoate methanesulfonate salt, Sigma-Aldrich, Merck KGaA, Darmstadt, Germany) and implanted with TT cells [60], as previously described [61]. Briefly, TT cells were labeled with a blue fluorescent viable dye (CellTracker Blue CMAC (7-amino-4-Chloromethylcoumarin, ThermoFisher, Waltham, MA, USA) following the manufacturer’s instructions, resuspended with PBS, and about 500–1000 stained cells per embryo were grafted into the subperidermal space of 48 hpf *Tg(fli1a:EGFP)^y1^* embryos, close to the SIV (subintestinal vein) plexus. As control of the implantation, we considered embryos injected with only PBS. Injections were performed by a micro-injector FemtoJet (Eppendorf, Hamburg, Germany) equipped with a micromanipulator InjectMan NI 2 (Eppendorf, Hamburg, Germany). Implanted embryos and controls were raised at 32 °C, a compromise temperature between 28 °C (the optimal temperature for zebrafish development and maintenance) and 37 °C (the optimal temperature for mammalian cell growth and metabolism).

**Figure 9:**
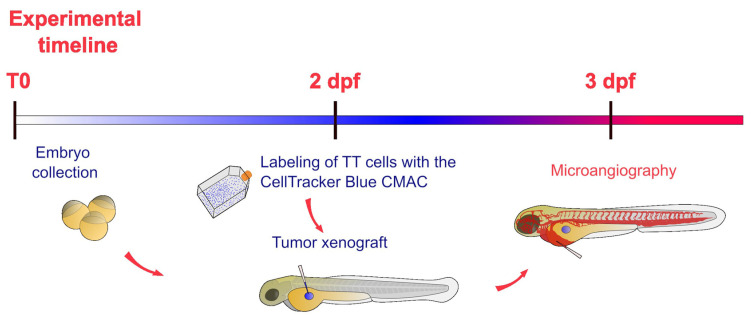
Schematic representation of the experimental timeline of experiments in zebrafish embryos. After the collection (T0), zebrafish embryos were incubated at 28 °C up to 2 dpf. At this stage labeled TT cells were implanted in embryos. After the xenograft, embryos were incubated at 32 °C for 24 h. The day after, microangiography assays were performed on 3 dpf embryos.

At 3 dpf, 24 h after the implantation of MTC cells, embryos were anesthetized and placed in 1% agarose-modified Petri dishes to perform the injection into the blood flow at the level of the sinus venosus (SV) of 4 nl of dextran tetramethylrhodamine (2,000,000 MW, ThermoFisher, Waltham, MA, USA). We performed microangiography assays on xenografted and control embryos. A schematic representation of the experimental plan in zebrafish is reported in Figure 9.

Immediately after the microangiography, the sample was observed with a fluorescence stereomicroscope to evaluate the correct spread of dextran rhodamine along the entire vascular tree. Some representative images have been acquired using a Leica M205 FA microscope equipped with a Leica DFC450 C digital camera and LAS V4.2 software (Leica Microsystems, Wetzlar, Germany). The duration of the procedure was about 300 s.

### 4.7. Zebrafish Sample Preparation for SPIM Analyses

To optimize the index matching for the experiments and to facilitate the positioning of the sample in the immersion chamber, we prepared an observation chamber by inserting and gluing 500 μm diameter FEP tubes inside glass capillaries with 800 μm internal diameter. A 1 mL syringe was glued to the other end of the capillary to aspirate the embryos.

Zebrafish embryos were anesthetized as described above and immersed in 0.1% agarose solution. They were then aspirated with the syringe until they were positioned about halfway along the length of the FEP tube. A small amount of 0.016% of tricaine was also added to the agarose solution to prevent the embryo from moving during the measurement. In addition, since the capillary is kept in a vertical position during the experiments, we let an air bubble form in the bottom part of the FEP tube, which acted as a cap and prevented the fish escaping from the tube.

### 4.8. Spatio-Temporal Diffusion

To study the dynamics of the spreading of the fluorophore, quantified by its diffusion coefficient, we directly exploit the Fick’s second diffusion law,

(2) $$\frac{\partial C\left(x,t\right)}{\partial t}=\frac{\partial }{\partial x}\left(D\frac{\partial C\left(x,t\right)}{\partial x}\right)\;\;$$

where $C\left(x,t\right)$ is the concentration of the diffusing fluorescent species. The analytical solution of Equation (2) can be written once the boundary conditions and the symmetry of the problem are defined. We assume that the experimental conditions (fluorophores in a vessel) are approximated by a situation where a fixed quantity of fluorescent solution is inserted in a semi-infinite bar. This situation corresponds to diffusion in a cylindrically symmetric system whose solution is obtained, in cylindrical coordinates [62], as the superposition of Bessel functions of the first kind. To simplify the problem and gain insights into the general behavior of diffusion, we use the solution in spherical coordinates, which can be expressed as a straightforward Gaussian function of space and time.

With the following initial conditions,

(3) $$\int_{V}C\left(x,t\right)dx=B;\;\;C\left(x,t=0\right)=0$$

we can write in this case:

(4) $$C\left(x,t\right)=\frac{B}{\sqrt{\pi D\left(t+t_{0}\right)}}{exp}^{\left(\frac{-x^{2}}{4D\left(t+t_{0}\right)}\right)}$$

The initial time ($t_{0\;}$) is defined as the delay between the injection and the start of the signal recording. The time trend of the concentration measured at a fixed observation position (identified by the coordinate x = $R_{0}$) is generally a bell-like curve skewed towards short times and with a slow tail at large times (see Figure A1 in Appendix A). The trend of concentration over time remains unchanged for the product Dt. The function’s shape stays consistent across different diffusion coefficient values, with the only difference being a dilation of the time axis. The trend, when observed from the very first time points after injection, would be a smooth sigmoidal increase towards a maximum, followed by a shallow decrease with time for times larger than $t_{max}=\frac{R_{0}^{2}}{2D}$. At very short distances (and times) from the injection point (time), we also expect a pressure effect on the propagation of the molecule through the medium. The injection implies an increase of pressure at the site of the injection, and this will push the injected molecules through the pores with a limiting speed. The result would be a linear increase of the concentration (and the fluorescence signal) as a function of time that will fade out by increasing the distance between the observation and the injection point.

To follow the diffusion of the fluorescent dye inside the zebrafish body, we acquired a stack of images versus time following the injection of the fluorescent dye. The fluorescence intensity profiles measured on the image time stacks at a fixed distance from the vessel were fitted to Equation (4) and the diffusion coefficient of the dye was determined as a fitting parameter. As a cross-check, we measured the velocity of the diffusing fluorescent dye at different distances from the injection point, by evaluating the time, $\tau_{\frac{1}{2}}$, at which the intensity halved its initial value. We found, as expected, a linear dependence of the time over the distance, and the angular coefficient represents the speed of the diffusing dye:

(5) $$v=\frac{R_{0}}{\tau_{\frac{1}{2}}}$$

which can be related to the diffusion coefficient by the relation (see Appendix A):

(6) $$\sqrt{\frac{4D}{\tau_{\frac{1}{2}}}}=vf\left(D\right)\;\;\;\;\;\;\;\;\;or\;R_{0}^{2}=\frac{4D\tau_{\frac{1}{2}}}{f(D)}$$

In Equation (6), f(D) = 0.013 D^2^ − 0.51 D + 8.14, and D is expressed in units of $\frac{{\mu m}^{2}}{s}$. It is worth noting that in the range of diffusion coefficients 1≤D≤20, the function $f\left(D\right)≃4$.

## Figures and Tables

**Table 1:** Diffusion coefficients of dextran rhodamine in agarose gels.

	D (μm^2^/s)	Pore size [nm] ^a^
1% Agarose	18.1 ± 0.2	140
2% Agarose	8.9 ± 0.1	60
5% Agarose	2.6 ± 0.1	16

Table 1 Diffusion coefficient, D, of dextran ($2\times 10^{6}\;MW$) rhodamine labeled versus agarose concentration, as derived from the global fitting of the diffusion curves in Figure 1 to Equation (4). (a): the last column reports the values derived from ref. [42].

## Data Availability

The original data presented in the study are openly available in Zenodo at https://doi.org/10.5281/zenodo.14016517.

## Appendix A

### Simulations of the Fluorescence Traces

Starting from the equation of diffusion, we can derive the analytical trend of the concentration upon the injection at t = $\;t_{0}$, of a concentrated fluorophore suspension in a specific 3D volume in x =$\;R_{0}$, as when observed at x = R with respect to the edge of the field of view (FOV) of the image:

(A1) $$C\left(R,t\right)=\frac{B}{\sqrt{\pi D\left(t+t_{0}\right)}}{exp}^{\left(\frac{-x^{2}}{4D\left(t+t_{0}\right)}\right)}$$

First of all, we consider the concentration as a function of time, leaving R as a parameter. This function has a bell-like shape with maximum at $t_{max}=\frac{{\left(R+R_{0}\right)}^{2}}{\left(2D\right)}$ and maximum amplitude $C_{max}=\frac{B}{D}\sqrt{\frac{2}{e}}$. Simulations of the trend for increasing values of the observation volume at D = $1\frac{\mu m^{2}}{s}$ and D = $0.1\frac{\mu m^{2}}{s}$ are reported in Figure A1.

As can be judged by Figure A1, the trend of the concentration upon time is invariant for the product Dt. The shape of the function is the same for different values of the diffusion coefficient and the only change is a dilation of the time axis. The trend, when observed from the very first time points after injection, is a smooth sigmoidal increase towards a maximum, followed by a shallow decrease with time for times larger than $t_{max}=\frac{{\left(R+R_{0}\right)}^{2}}{\left(2D\right)}$.

The trend reproduced in Figure A1 is similar to the ones observed for the diffusion of the fluorophores in agarose gels as reported in Figure 1.

The experiments undertaken on zebrafish embryos were performed in such a way that a delay of about 1800 s was present between the injection and the start of the observations.

The trend observed in Figure 4 is indeed reproduced well by the simulations when the data are reported with a shift of the time scale of about 2000 s, as in Figure A2.

Finally, we observe that there is a relation between the position of the observation region of interest (ROI) and the half time of the decay from the maximum of the concentration curve. We define the following ratio between the position of the ROI, R and the half maximum decay time (measured with respect to the maximum time, $t_{max}=\frac{R^{2}}{\left(2D\right)}$):

(A2) $$v_{R}=\frac{R}{\tau_{\frac{1}{2}}}$$

This parameter is a measure of the spreading of the dye in the medium. It has the units of speed and can be put in relation with a second independent measure of the spread of the concentration wave, again with the units of speed:

(A3) $$v_{D}=\sqrt{\frac{4D}{\tau_{\frac{1}{2}}}}=vf\left(D\right)$$

From the simulations reported in Figure A3, we can derive that the relation between $v_{R}$ and $v_{D}$ can be written as:

(A4) $$v_{D}=v_{R}f\left(D\right)$$

Equations (A3) and (A4) also account for a diffusive trend of the type:

(A5) $$\Delta r^{2}=4\frac{D}{f\left(D\right)}\tau_{\frac{1}{2}}$$

In Equations (A4) and (A5), f(D) = 0.013 D^2^ − 0.51 D + 8.14, and D is expressed in units of $\frac{\mu m^{2}}{s}$.

The quadratic dependence of Equation (A5) can be easily confirmed by numerical simulations as reported in Figure A4a.

However, the estimate of the half-decay time at the experimental level is affected by an intrinsic uncertainty concerning the delay of the start of the acquisition with respect to the injection time. Therefore, the experimental estimate of the half-decay time must be taken as an effective value $\tau_{\frac{1}{2}}^{*}$. We can study the relationship between $\tau_{\frac{1}{2}}$ and $\tau_{\frac{1}{2}}^{*}$ for different values of the diffusion coefficient and the delay time $t_{0}$. This study can be summarized in Figure A4b, where a direct comparison of the numerically simulated values of $\tau_{\frac{1}{2}}$ is compared to experimental values for the control (open squares) and xenografted (filled squares) zebrafish.
